# Loureirin B downregulates osteoclast differentiation of bone marrow macrophages by targeting the MAPK signaling pathway

**DOI:** 10.1038/s41598-022-18287-5

**Published:** 2022-08-23

**Authors:** Jiahao Zhang, Liang Mo, Haoran Huang, Jiake Xu, Yinuo Fan, Weifeng Li, Haibin Wang, Chi Zhou, Hanjun Fang, Wei He, Zhenqiu Chen, Yuhao Liu

**Affiliations:** 1 The First Affiliated Hospital, Guangzhou University of Chinese Medicine, Guangzhou, China; 2 School of Biomedical Sciences, University of Western Australia, Perth, Australia; 3 Guangdong Research Institute of Orthopedics & Traumatology of Chinese Medicine, Guangzhou, China

**Keywords:** Cell biology, Stem cells

## Abstract

Excessive absorption of osteoclasts will break the balance between osteoclasts and osteoblasts, leading to bone loss, decreased bone density, and increased bone fragility. We have shown that Loureirin B (LrB) can inhibit osteoclasts. In this study, we demonstrated the targeting-inhibitory mechanism of LrB acting on osteoclast precursor. Using SPR, HPLC and MALDI-TOF-MS to capture and analyze the target protein of Loureirin B in bone marrow macrophages (BMMs), we used this method to detect all target proteins that LrB acts on BMMs, and analyzed the distribution and enrichment rate of the target protein by DAVID enrichment analysis. Ledock molecular docking was used to detect the binding of LrB. We used Western Blot for verification. The target proteins of LrB acting on BMMs were Serpine1, Atp6ap1, Dvl1, Rhd, Fzd2, MAPK1, MAP2K2, MAPK3 and so on. MAPK1, MAP2K2 and MAPK3 were the most relevant. LrB treatment attenuated the expression of phosphorylated JNK and p38 kinases of the MAPK signaling pathway. Our research further confirmed that LrB affects the MAPK signaling pathway in BMMs, thereby inhibiting the differentiation of BMMs into osteoclasts. This discovery can confirm the mechanism by which LrB acts on BMMs.

## Introduction

Bone is an important tissue of our bodies that provides support and protection for soft tissues, regulates mineral homeostasis, and maintains the microenvironment of the medullary cavity^1,2^. The processes of bone conformation and remodeling are coordinated by different cells, such as bone lining cells, osteoclasts, osteoblasts and osteocytes^3,4^. Osteoclasts, which differentiate from the macrophage lineage, have the ability to dissolve bone^5^. Normal bone metabolism is a dynamic balancing process of continuous circulation of bone formation and bone resorption^6,7^. In this process, the senescent bone is absorbed and removed by osteoclasts. Osteoblasts are incorporated into the newly formed osteoid and eventually become mature bone cells embedded in calcified bone^8,9^. Some bone diseases, such as osteoporosis, are caused by hyperactivated osteoclast resorption than osteoblastogenesis. Long-term excessive bone resorption affects the balance between osteoblasts and osteoclasts, leading to bone loss, bone density reduction, bone fragility, and a series of diseases caused by increased bone resorption^10^.

Many studies have shown that the proliferation and differentiation of osteoclasts is completed by the comprehensive actions of multiple signaling pathways. The RANKL/RANK/OPG signaling pathway and NF-κB signaling pathway promote the formation of osteoclasts^11,12^. MAPK/ERK signaling pathway, M-CSF signaling pathway, and Ca^2+^ signaling pathway are involved in the process of osteoclast differentiation^13–15^. The Src and Akt signaling pathways have an impact on the activity of osteoclasts^16,17^.

Loureirin B (LrB), isolated from a traditional herb named Sanguis draxonis, is one of more than 12 kinds of active components, and it has been used widely to treat diseases^18^. Previous studies showed that LrB has biological effects on inhibiting the proliferation and promoting the apoptosis of hepatic stellate cells^19^. It can also promote insulin secretion by Ins-1 cells through GLP-1R^20^. Our previous study showed that LrB inhibits osteoclastogenesis, bone resorption, and osteoclast specific gene expression by blocking NFATc1 translocation and MAPK-NFAT signaling pathways. This indicates that LrB prevents OVX-induced osteoporosis and preserves bone volume by repressing osteoclast activity and function^21^. However, the mechanism by which LrB acts on bone marrow macrophages (BMMs), which are the precursors of osteoclasts, has not yet been elucidated. In view of the effect of LrB on BMMs, this study focused on the target proteins in osteoclastic pathways through proteomics to clarify the mechanism of LrB acting on BMMs.

## Methods

### Surface plasmon resonance (SPR) technique

#### Sample preparation

The 6-week mice were sacrificed by cervical dislocation and placed in 75% alcohol for disinfection. After taking out the bilateral femur and tibia, the bone marrow macrophages were extracted from the medullary cavity and grown in a culture medium (25 ng/mL M-CSF, 100 U/ml of penicillin/streptomycin and 10% FBS in α-MEM, without RANKL) in T75 flasks. Then, the BMMs were trypsinized for 3 to 4 min and centrifuged (25 °C, 1000 r, 5 min), and cryopreservation solution (Fatal bovine serum (FBS): Dimethyl sulfoxide (DMSO) = 9:1) was added for storage at − 80 °C. Loureirin B powder was prepared to a working concentration of 10 mM with DMSO. All animal procedures were approved by the Experimental Animal Ethics Committee of Guangzhou University of Chinese Medicine. All methods mentioned in the article were in accordance with ARRIVE guidelines, and have been confirmed in accordance with relevant guidelines and regulations.

#### Cell lysis treatment

The cell sample was thawed from the refrigerator, and centrifuged briefly to concentrate the sample at the bottom of the EP tube. The cell sample was fully shaken with 120 μL PBS and Halt Protease Inhibitor Cocktail (100x, Thermo Fisher) at a final concentration of 1% and mixed with BWLS-17 Lysis Solution. Then, the cell samples were processed according to the SOP of the "Syringe Jet Lysis Method", centrifuged (4 °C, 16,000*g*, 10 min), aliquoted and stored at − 40 °C.

Before the test, the sample was thawed and centrifuged (4 °C, 16,000*g*, 10 min). The supernatant was taken for concentration determination (Thermo Fisher BCA Protein Assay Kit), and adjusted with 1× stock solution to a final concentration of 200 µg/mL.

#### Fabrication of photo-crosslinking sensor chip

The cell lysis solution was adhered on one 50 × 50-well plate. After the temperature of the photo-crosslinking sensor chip was reduced to room temperature, the Loureirin B solution was placed on the designated area on the 3D photo-crosslinking sensor chip by high-throughput array printer. When arrayed printing by the BioDot™ 1520 array printer, it must be at a pressure of 1.06 ATMs room were strictly protected from light and filled with nitrogen. To control the same sample dose, a two-needle system must be adopted, with 280 µm of the distance of every spot and 180 µm of their diameter, to add the solution five-times to 312. 5 µL of the dose in every spot. After the chip became dry, it was removed into the UV Spectroirradiator (1020, Amersham Life Science), with 365 nm wavelength and 1.20 ATM nitrogen pressure, to begin the light-crosslinked reaction.

#### Chip washing

After the photo-crosslinking reactions, the chip was shaken and washed with dimethyl formamide (DMF), absolute ethanol and ultrapure water on a shaker for 15 min. When the chip was dry blown with nitrogen in a clean laboratory, it was put on cover, marked and stored at − 20 °C.

#### SPR capture online

After the chip was placed in the SPR Biochip Analysis System, the experimental baseline was regulated, and circulate regeneration fluid (glycine·HCl, pH = 2.0), whose rate was 3 µL/s and duration was 300 s, was used to regenerate the chip three- times in carrier buffer (PBST; pH = 7.4, 0.05% Tween-20). Then the chip was blocked in carrier buffer with 100 µg/L bovine serum albumin (BSA) at a rate of 3 µL/s and duration of 300 s. Finally, the surface of the chip was also regulated with the same regeneration fluid.

To maintain balance in the system, the carrier buffer (PBST; pH = 7.4) was added at a rate of 2 µL/s and a duration of 260 s. Additionally, the bone marrow macrophage sample was attached and cleaned at the same rate and duration in the carrier buffer. Then the chip was put in trypsin to hydrolyze after all the tests finished.

### Liquid chromatography–mass spectrometry (LC–MS)

#### Enzyme digestion of the chip

The chip was mixed with 10 nM dl-dithiothreitol and placed at 56 °C for 1 h. When the chip finished its deoxidation, it was mixed with 55 mM iodoacetamide solution in a black room for 45 min. After aspirating the buffer in the chip, 30 µL 0. 25 M tetraethyl ammonium bromide (TEAB) was added to clean the chip three times, mixed trypsin (1 µg/µL, stored in 50 nM acetic acid) and protein solution at a ratio of trypsin to protein solution of 1:20, and placed in the mixed solution at 37 °C overnight. Then the sample was mixed with the trypsin solution at the same proportion and incubated at 37 °C for 4 h. When the proteins had completely digested, the solution was removed from the EP tube, added 30 µL of 0.5 M TEAB was added to mix the filtrate, and the tube was dried under a vacuum.

#### Identification of the LC–MS proteins

Ten microliters of HPLC mobile phase A (pH = 2.5, adjusted by chromatographic pure formic acid), which contained 5% acetonitrile (ACN) and 0.1% aqueous formic acid, was added to solute the dry sample of peptide fragments. The peptide fragment solution was added at a rate of 10 µL/min for 3 min. The peptide fragments were captured directly by a peptide capture column (100 μm × 2.0 cm Acclaim PepMap C18, Thermo Fisher), and the salt solution was turned into waste liquid. Then, the machine was switched to a valve to link the capture column and the analysis column, the nano pump was started up for reversed-phase separation and the peptide were detected fragments by mass spectrum acquisition online. The process of reverse-phase took 60 min. In this process, the density of HPLC mobile phase B (pH = 2.5, containing 90% acetonitrile (ACN) and 0.1% aqueous formic acid) increased from 2 to 45%, the rate was 300 nL/min, and the temperature of the column was 40 °C.

#### Mass spectrum analysis

When the ion source spray voltage is 2.0 kV and the heating capillary of the mass spectrometer is set to 250 °C, the data-dependent mode is adopted to automatically switch the acquisition between MS and MS/MS. The full scan MS scanned by Orbitrap was set to 90 min of the scan time, m/z 350 1600 of the scan range, and 70,000 (m/z 200 spots) of the resolution. A quadrupole was used to screen the precursor ions, and then higher energy C trap dissociation (HCD) was used to fragment the precursor ions that met the fragmentation conditions of cascade MS/MS and scanned with Orbitrap. The scanning resolution was set to 17,500, and the scanning range was automatically controlled according to the mass-to-charge ratio of the precursor ion. The top 15 ions in intensity were scanned by the MS/MS scan which had 27% collision energy in high purity nitrogen. MS data was collected by Xcalibur Software (Thermo Scientific version 2.4.5).

#### Identification of proteins

The MS data were searched by the Mascot algorithm using Proteome Discoverer (Thermo Fisher Scientific, version 1.7) analysis software. The search database was the UniProtKB/Swiss Prot protein database. To reduce false positive results, it was added that a decoy database contained all protein reversal sequences. This process could identify all the proteins linked to target proteins.

### DAVID enrichment analysis

All the target proteins obtained by proteomics were entered into DAVID Bioinformatics Resources 6.8 (DAVID Functional Annotation Bioinformatics Microarray Analysis (ncifcrf.gov); https://david.ncifcrf.gov/tools.jsp). The selected identifier was OFFICIAL_GENE_SYMBLE and the selected species was mouse. Then all the results were analyzed by Gene Ontology (GO) enrichment analysis and Kyoto Encyclopedia of Genes and Genomes (KEGG) enrichment analysis. They included the proteins of biological process, cell component and molecule function, and the proteins of all the related pathways.

### Molecular docking

The top three proteins related to Loureirin B could be determined according to the analysis of the results of the proteins. The structures were obtained from the PDB website (RCSB PDB: Homepage; https://www1.rcsb.org/). The minimized energy of the molecular structure of Loureirin B was calculated in Chem3D. The energy of the proteins combined with Loureirin B could be obtained by Ledock and the images of the proteins combined with Loureirin B were acquired by PyMOL.

### Western blot

The cryopreserved cells were resuspended and cultured in T75 flasks. For the short-term Western blotting assay, BMMs were seeded in 6-well plates and cultured with culture medium until they reached 90% confluence. Total cellular proteins were extracted using RIPA lysis buffer. SDS-PAGE was used to separate proteins, and the protein bands were transferred to a nitrocellulose membrane. After 2 h of blocking with 5% skim milk, MAPK primary antibodies were added onto the precisely-cut membranes and then hybridized overnight at 4 °C. The corresponding secondary antibodies were then administered and incubated for 1 h. Antibody reactions were detected using a Western Lightning Ultra Detection Kit, and images were taken by the FujiFilm LAS-4000 Gel Documentation System and its associated software.

## Results

### Target proteins

**Figure 1:**
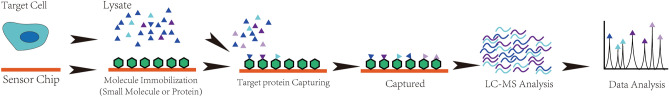
The diagrams of process and mechanism of the SPR, HPLC and TOF–MS.

**Figure 2:**
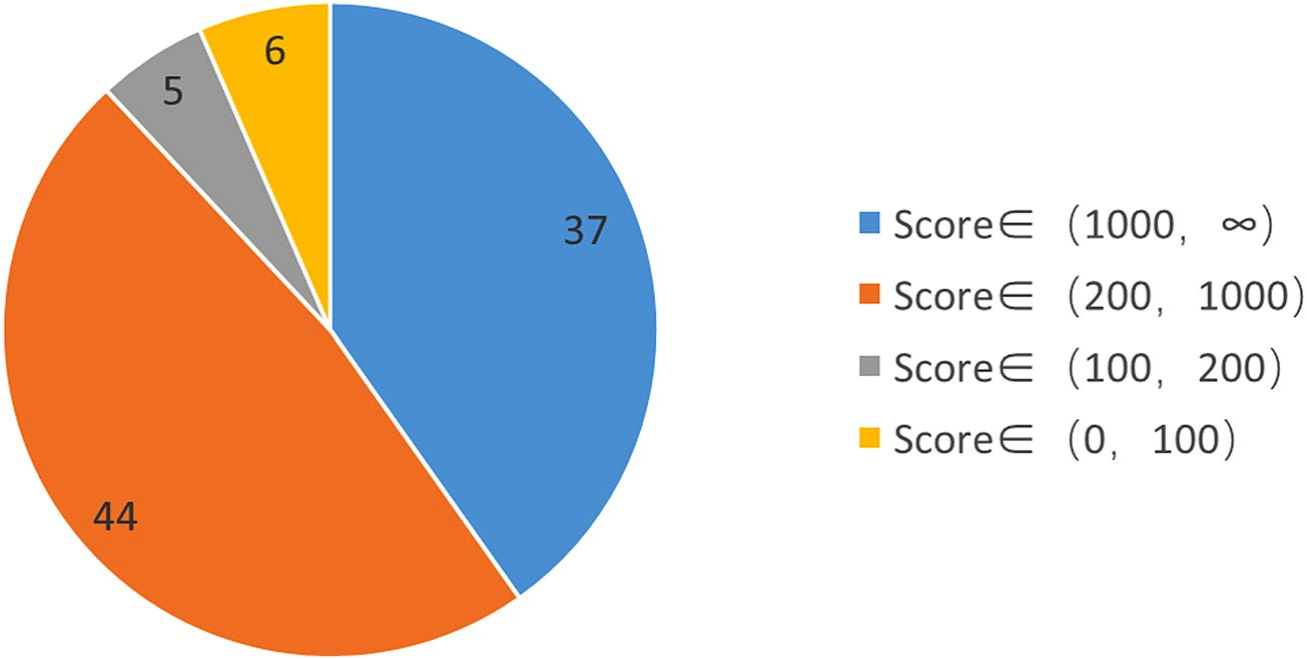
The score of the target protein that LrB acts on BMMs and the proportion of different score segments. (1) Targets with Score > 1000: high-affinity binding, characterized by fast binding and high affinity. Such target proteins are usually related to acute drug efficacy, acute toxicity and drug metabolism; (2) Targets with 200 < Score < 1000 Point: medium-affinity binding, characterized by binding speed and medium affinity, such targets are usually related to chronic drug effects; (3) Targets with 100 < Score < 200: low-affinity binding, characterized by slow binding and relatively low affinity Low, such targets are usually related to drug delivery, blood drug concentration maintenance, and bypass drug efficacy.

Through the SPR, HPLC and TOF–MS (Fig. 1), 92 proteins targeted to loureirin B was obtained, six of which had a score less than 100 and were excluded. The score of 37 target proteins in 86 included proteins was more than 1000, which means that this kind of protein had high affinity and quick binding speed (Serpine1, Atp6ap1, Dvl1, Rhd, Fzd2, MAPK1, MAP2K2, MAPK3 and so on) (Table 1). This mean that this kind of protein had moderate affinity and binding speed, with a score of 44 from 200 to 1000 (Nqo2, Atrn, Tdp1, Npc1, Kdm4d and so on). Five of them between 100 and 200 mean that the affinity and binding speed were low (Ttr, Celsr2, Plat, Uqcrfs1, Dpt) (Fig. 2).

**Table 1:** The scores of the part of the target proteins of LrB acting on BMMs.

	Protein names	Abbreviation	Score
1	Plasminogen activator inhibitor 1	Serpine1	1834.12
2	V-type proton ATPase subunit S1	Atp6ap1	1756.01
3	Segment polarity protein dishevelled homolog DVL-1	Dvl1	1708.94
4	Blood group Rh(D) polypeptide	Rhd	1705.21
5	Frizzled	Fzd2	1699.02
6	Mitogen-activated protein kinase 1	Mapk1	1424.77
7	Mitogen-activated protein kinase 3	Mapk3	1209.99
8	Dual specificity mitogen-activated protein kinase kinase	Map2k2	1033.68

### Gene Ontology (GO) enrichment analysis results

**Figure 3:**
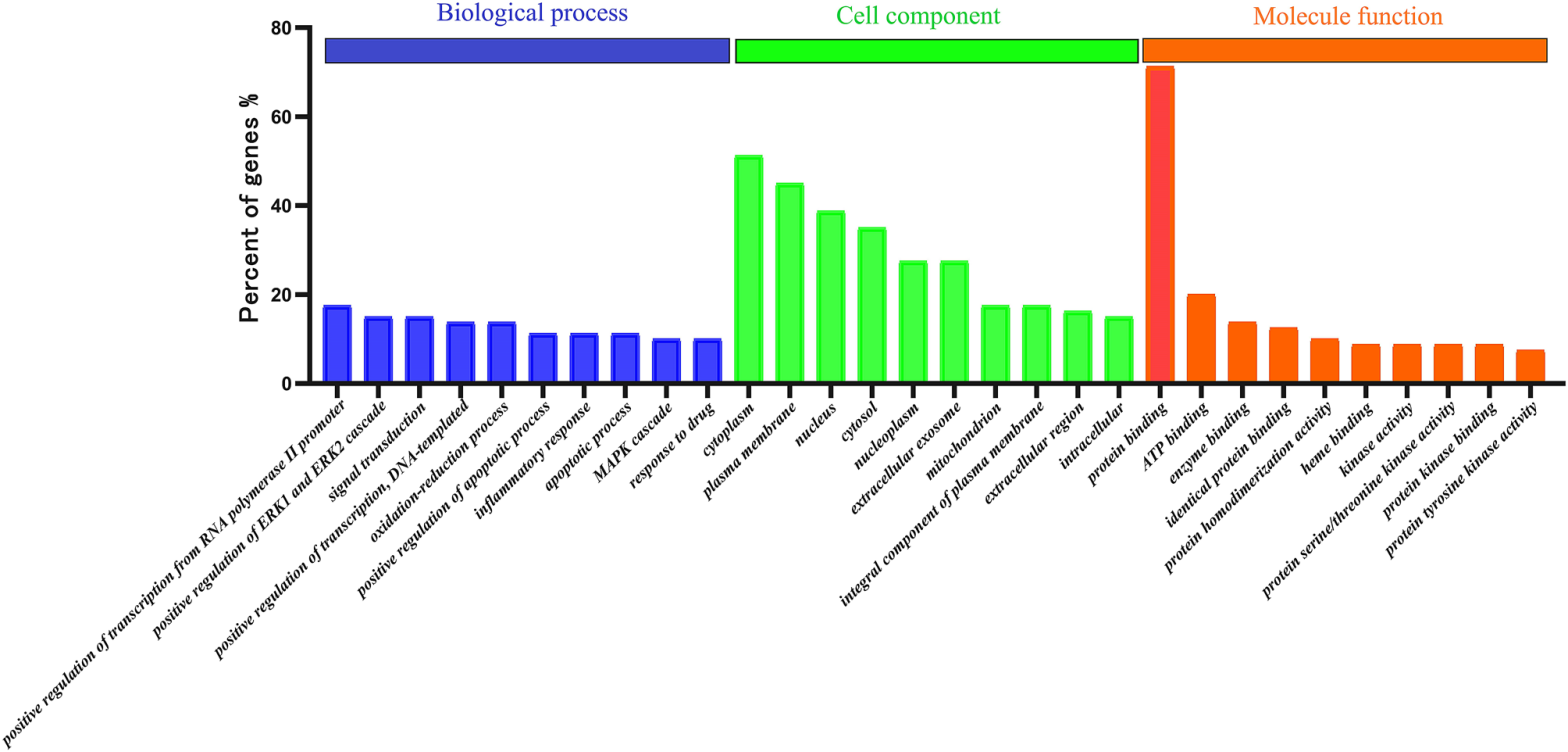
The enrichment ratios of target proteins in biological process, cell composition and molecular function in biological function obtained by GO enrichment analysis.

The results of Gene Ontology (GO) enrichment analysis showed that the proteins that took part in the biological process, were mainly positive regulation of transcription from the RNA polymerase II promoter, positive regulation of the ERK1 and ERK2 cascades and signal transduction. The cell components they mainly took part in were cytoplasm, plasma membrane and nucleus. The molecule functions were protein binding, ATP binding and enzyme binding (Fig. 3).

### Kyoto Encyclopedia of Genes and Genomes (KEGG) enrichment analysis results

**Figure 4:**
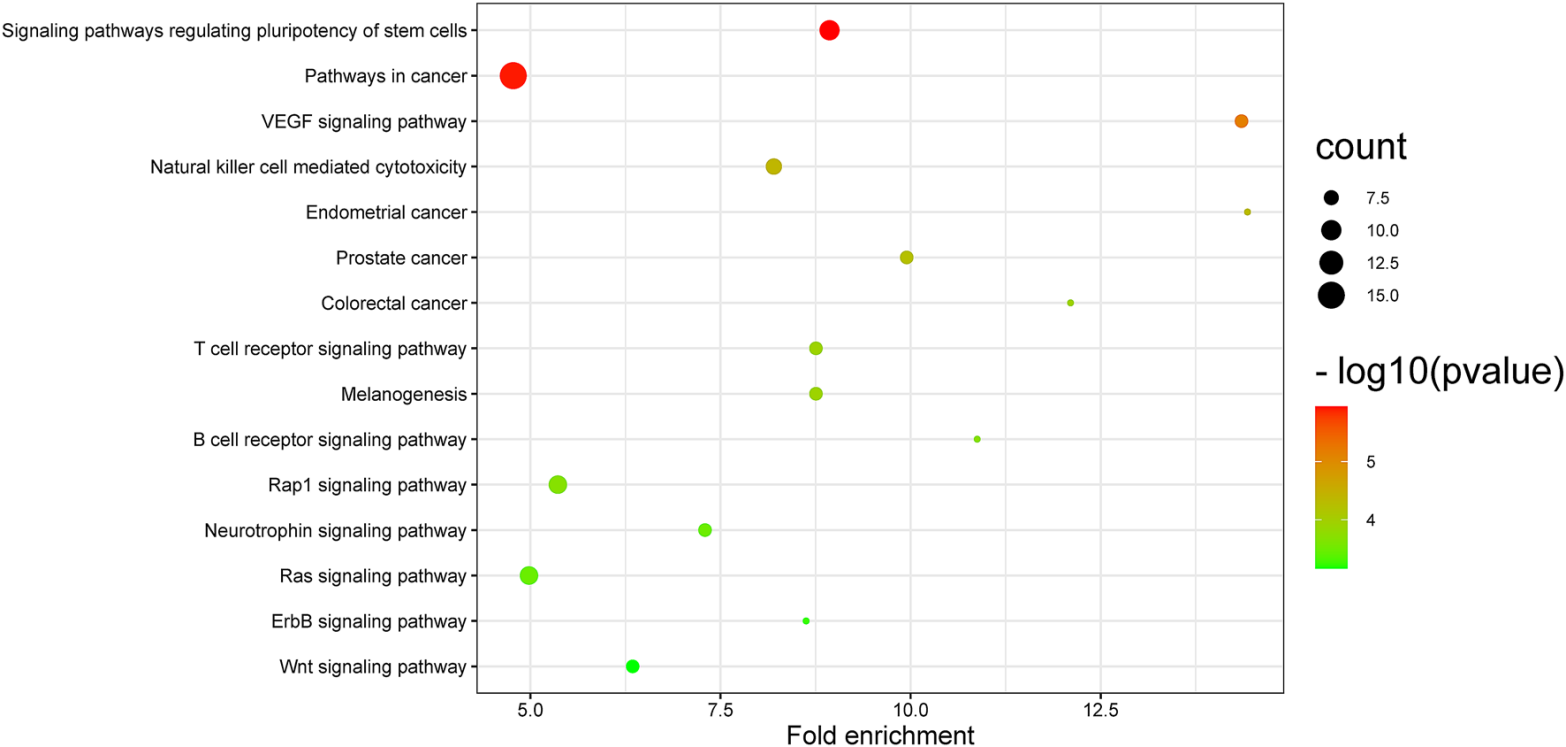
The top 15 signaling pathways with the highest enrichment ratio. The size of the circle represents the number of enriched target proteins. The p value represents the significant degree of enrichment of the target protein.

**Figure 5:**
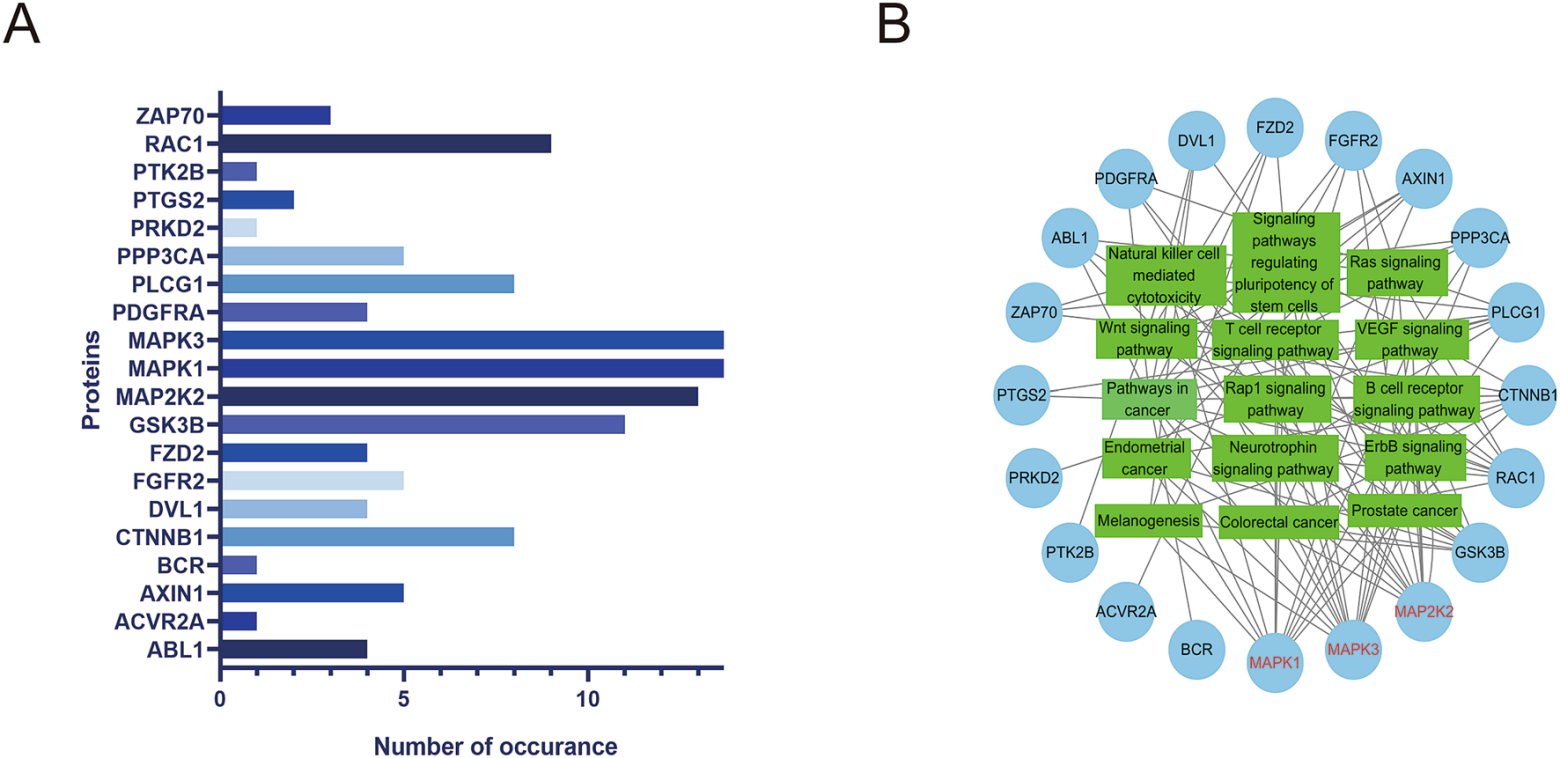
The numbers of target proteins of the top 15 signaling pathways with the highest enrichment ratio. (**A**) The number of occurrences of each target protein in the top 15 signaling pathways. (**B**) The circles represent the target proteins, and the squares represent the signaling pathways. The number of lines between the square and the circle represents the number of occurrences.

The KEGG results showed the signaling pathways by which Loureirin B affected BMMs. The pathways were sorted by fold enrichment and included pathways in cancer, signaling pathways regulating pluripotency of stem cells, the Rap1 signaling pathway and the Ras signaling pathway (Fig. 4). In the top 15 of the most relevant pathways, the number of MAP2K2 occurrences was 13, and the numbers of MAPK1 and MAPK3 occurrences were 14 (Fig. 5).

### Molecular docking results

**Figure 6:**
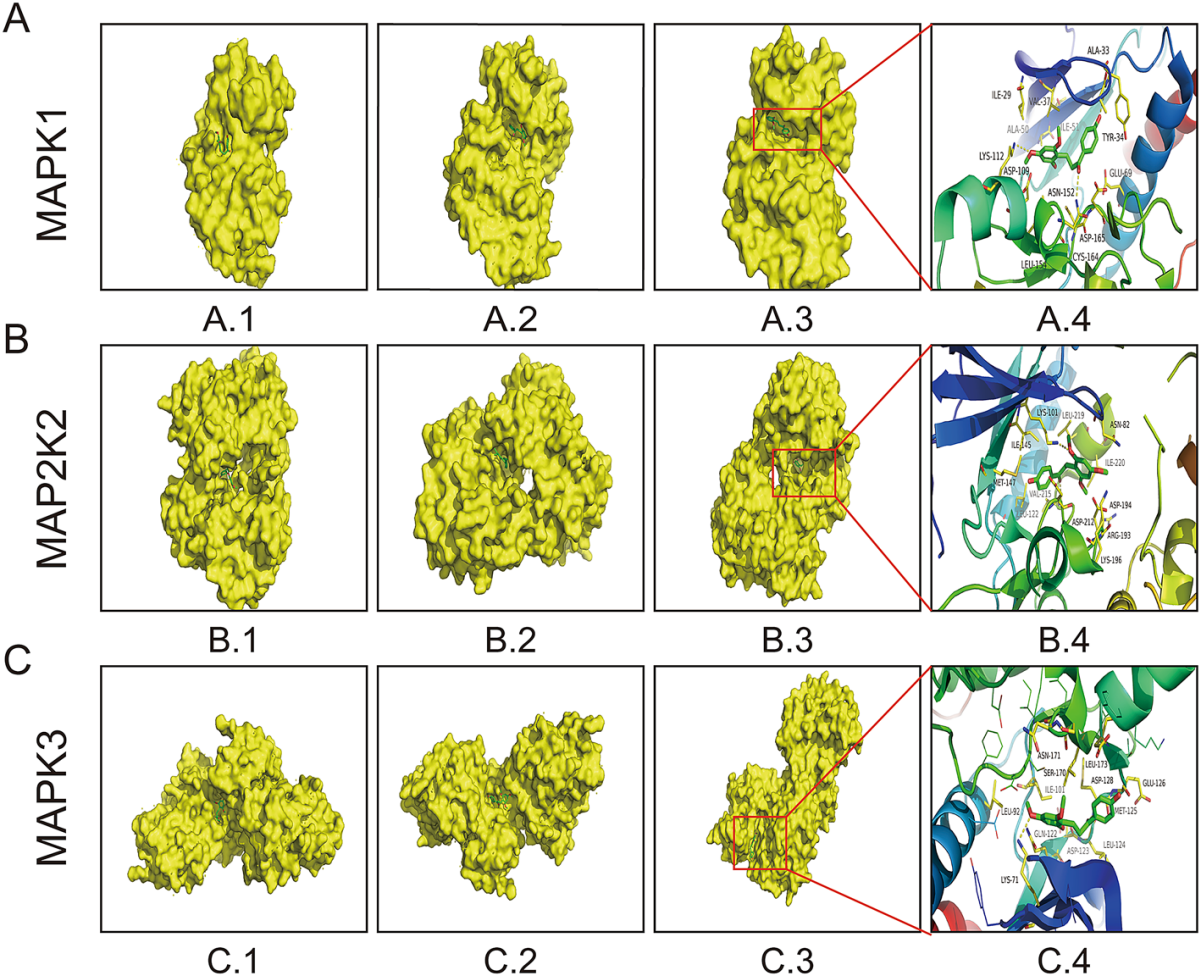
The binding sites of LrB and MAPK1, MAP2K2 and MAPK3. (A.3–C.3) The sites with the highest binding capacity of LrB to MAPK1, MAP2K2 and MAPK3. (A.4–C.4) The molecular structures of the sites with the highest binding capacity of LrB to MAPK1, MAP2K2 and MAPK3.

The results of molecular docking showed the energies and combined structures of Loureirin B combined with MAPK1, MAP2K2 and MAPK3 (Fig. 6). The energies of the three MAPK1 sites were − 4.98 (A.1), − 5.97 (A.2) and − 6.37 (A.3) kcal/mol. The energies of MAP2K2 were − 4.96 (B.1), − 4.80 (B.2) and − 6.47 (B.3) kcal/mol, and those of MAPK3 were − 4.84 (C.1), − 4.83 (C.2) and − 5.85 (C.3) kcal/mol (Table 2).

**Table 2:** The binding energies of LrB to different sites in MAPK1, MAP2K2 and MAPK3.

Molecule	Site	Energy (kcal/mol)
MAPK1	A.1	− 4.98
	A.2	− 5.97
	A.3	− 6.37
MAP2K2	B.1	− 4.96
	B.2	− 4.80
	B.3	− 6.47
MAPK3	C.1	− 4.84
	C.2	− 4.83
	C.3	− 5.85

The lower the binding energy, the better the binding ability. It represented the sites of LrB and MAPK1, MAP2K2 and MAPK3 were best that the binding energies of A.3, B.3 and C.3 were lowest in each group.

### Western blot

**Figure 7:**
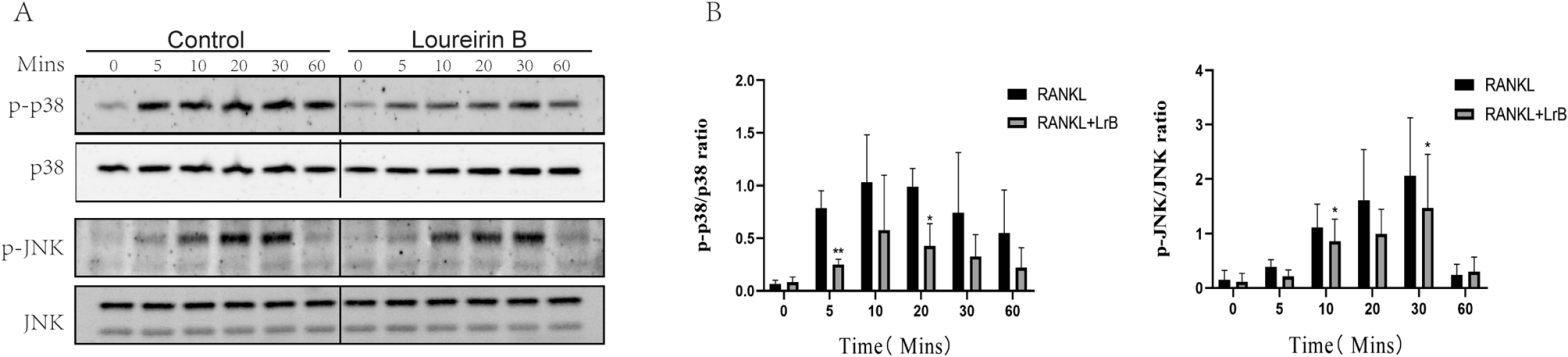
LrB inhibits MAPK and NFATc1 signaling pathways. LrB was used to pretreat the BMMs for 1 h followed by 0, 5, 10, 20, 30 and 60 min. Total cellular proteins were extracted using RIPA lysis buffer and cell lysates were analyzed by Western blotting using primary antibodies specific to p-p38, p38, p-JNK and JNK. The ratios of p-p38/p38 and p-JNK/JNK show the inhibitory effect of LrB on MAPK pathway signaling specific to p-p38 and p-JNK (n = 3).

To further study the mechanism by which LrB inhibits the differentiation of osteoclasts, we examined the impact of LrB on MAPK pathways. JNK and P38, phosphorylation of two MAPK family members, were downregulated. We found that LrB treatment inhibited phosphorylated JNK and P38 kinases (Fig. 7).

## Discussion

To maintain skeletal homeostasis throughout our lifespan, bone tissue must be constantly remodeled, which is regulated by osteoclasts and osteoblasts through their coupling activities. Osteoclasts, differentiated from bone marrow macrophages, have the function of absorbing bone and releasing mineralized substances. Osteoblasts are differentiated from bone marrow mesenchymal stem cells and play an important role in bone formation. The growth and stability of bones need to rely on the absorption and formation of the two functions to maintain a balance^22^. With increasing of age, the absorption function of osteoclasts gradually exceeds the formation function of osteoblasts, leading to the destruction of bone structure and bone loss. Studies have shown that the cause of osteoporosis is the destruction of bone structure, quality and integrity caused by increased bone resorption^23^. Sanguis draxonis, a traditional herbal medicine, contains a variety of active ingredients, such as flavans, triterpenes and so on. LrB is one of these active ingredients. At present, many studies have shown that Sanguis draxonis had anti-inflammatory, analgesic, antibacterial, antioxidant and anti-platelet aggregation^24^. And its side effects have not yet been reported. In this study, we proved that LrB acts on bone marrow macrophages through the MAPK pathway to regulate the differentiation of BMMs into osteoclasts.

Proteomics is a high-throughput screening technology that can be used to reveal biomarkers of life processes or major diseases, and to predict the targets and mechanisms of drug treatments^25^. Through this proteomics technology, the pathological mechanism related to the disease and the molecular mechanism of drug treatment are initially clarified, biomarkers of the disease and the potential targets of the drug are obtained, and new research directions are provided for the diagnosis and treatment of the disease^26^. The proteomics results showed that LrB mainly acts on BMMs through Serpine1, Atp6ap1, Dvl1, Rhd, Fzd2, MAPK1, MAP2K2, MAPK3 and so on. These target proteins could show that MAPK1, MAP2K2 and MAPK3 were the most relevant proteins for LrB to act on BMMs, as analyzed by GO enrichment analysis and KEGG enrichment analysis. The proteomics results also show that the scores of MAPK1, MAP2K2 and MAPK3 are all greater than 1000, which also shows that LrB has high affinity for MAPK family proteins. Therefore, the MAPK signaling pathway was considered to be one of the signaling pathways by which BMMs differentiate into osteoclasts.

The binding of RANKL and the receptor RANKL activates several key signaling pathways in cells, and promotes the proliferation and differentiation of BMMs into osteoclasts. Some of them are MAPK signaling pathways that are generated early by osteoclasts^27^. The MAPK signaling pathway including p38, ERK and JNK is stimulated to activate AP-1 to promote osteoclast formation^28^. P38 signal transduction is mainly involved in the differentiation of osteoclasts and osteoclastogenesis, rather than affecting the function of osteoclasts^29^. In osteoclastogenesis, osteoclast factors phosphorylate MAPKKKs. Then, phosphorylation of MAPKKKs, including MKK3 and MKK6, promotes osteoclastogenesis by inducing phosphorylation of p38^30,31^. Also, the phosphorylation of p38 induced through M-CSF-c-Fms signaling during macrophage development and the binding of RANKL to its cognate receptor RANK, thereby promotes the differentiation of osteoclasts^32^. JNK and ERK are essential for the formation of osteoclasts^33^. The ceramide 1-phosphate, which is reported to be be mitogenic for fibroblasts and acts as a lipid second messenger, is the cytokine from M-CSF stimulated bone marrow macrophages. It can mediate their proliferation via rapid phosphorylation of protein kinase B (also known as Akt) and JNK^34^ The previous studies had demostrated that some inflammatory cytokines, such as IL6, suppresses osteoclast differentiation through inhibition of JNK activation by upregulating the expression of DUSP1 and DUSP16 to dephosphorylate JNK^35^. The phosphorylation of ERK is also an important pathway for the formation of osteoclasts. The binding of M-CSF to its receptor c-Fms results in phosphorylation of c-Fms. Because of the phosphorylation site of the intracellular cytoplasmic tail of c-Fms interacts with growth factor receptor-binding protein-2, a stimulator of the Ras/Raf pathway, ERK is activated for enhancing osteoclast precursors proliferation and survival^36^. Besides, the phosphorylation of ERK also plays a role in maintaining cell polarity during bone resorption^37^. In conclusion, the phosphorylation of proteins of MAPK signaling pathway, including p38, JNK and ERK, is vital for the proliferation of BMMs and differentiation into osteoclasts. According to the Western Blot results, we found that LrB could inhibit the phosphorylation of p-38 and JNK in BMMs. We also found that it had no apparent effect of LrB on ERK, so did not present the image of western blot. Therefore, LrB has been confirmed to inhibit the phosphorylation of proteins of the MAPK signaling pathway, thereby affecting the differentiation of BMMs into osteoclasts. The MAPK signaling pathway also plays an important role in the proliferation and differentiation of vascular endothelial cells. Studies have shown that VEGF causes agonist protein remodeling in the tension fibers of endothelial cells through the Cdc42-SAPK2/p38-MAPK (MAP2K2) signaling pathway. Through the PLC-γ-PKC-Raf-MEK-MAPK signaling pathway, VEGF transmits signals to the nucleus to initiate DNA synthesis and promote the proliferation of endothelial cells^38^.

With age, the synthesis and secretion function of aging osteoblasts is reduced, which slows the rate of bone remodeling. The number of bone cells in the aging body will also decrease and increase apoptosis, resulting in bone resorption greater than bone production, and bone mass reduction^39^. We found that LrB reduced the phosphorylation of p-38 and JNK in the MAPK family, inhibited MAPK signal transduction, affected the proliferation and differentiation of BMMs into osteoclasts, and reduced bone resorption. LrB can quickly bind to MAPK family proteins and inhibit the MAPK signaling pathway, thereby reducing the differentiation of osteoclasts.

There are two limitations in this study. It verified that LrB could affect BMMs by inhibiting the phosphorylation of p38/MAPK and JNK/MAPK, but the inhibitory mechanism is still under unelucidated, even needs to be explored in animal models. Additionally, MAPK1 (ERK2), MAP2K2 (MEK2) and MAPK3 (ERK1) proteins were involved in Chip analysis, but it lacked of p38 and JNK proteins which phosphorylations were blocked by LrB (Supplementary Information).

## Supplementary Information

Supplementary Figures.

## Data Availability

The data that support the findings of this study are available, but restrictions apply to the availability of these data, which were used under license for the current study, and so are not publicly available. Data are however available from the authors upon reasonable request and with permission of us.
