# Supplementary material for: Loureirin B downregulates osteoclast differentiation of bone marrow macrophages by targeting the MAPK signaling pathway
Source: Sci Rep. 2022 Aug 23;12:14382. doi: 10.1038/s41598-022-18287-5 (PMC9399088; doi:10.1038/s41598-022-18287-5)

Supplementary Figures

Original images of Western blot

p38

p-p38

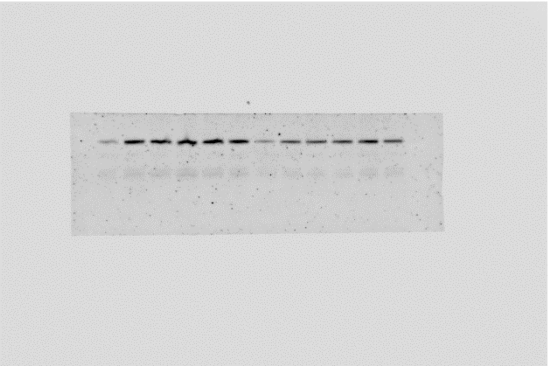

Band background of p38 and p-p38

p-p38

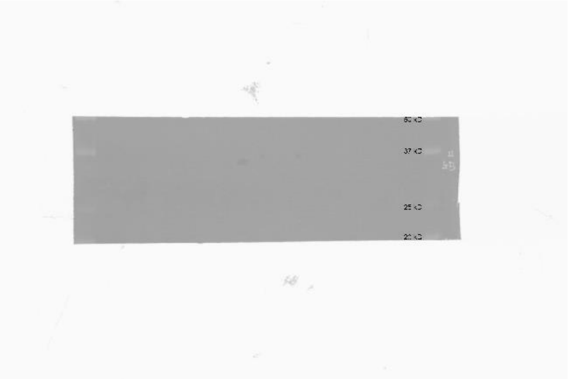

p-p38 Background

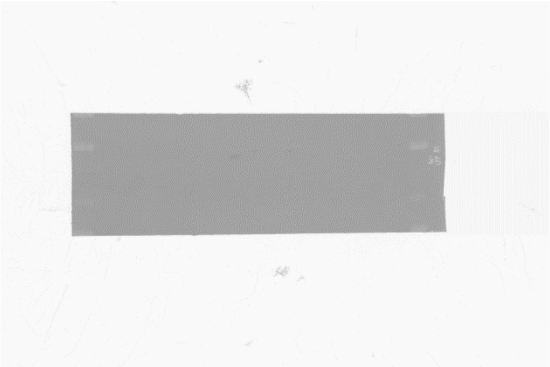

Original images of Western blot

JNK

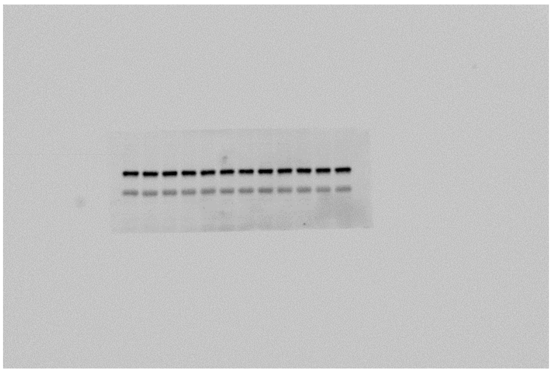

p-JNK

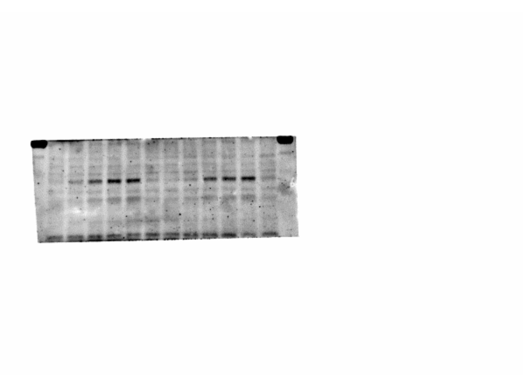

Band background of p38 and p-p38

p-JNK

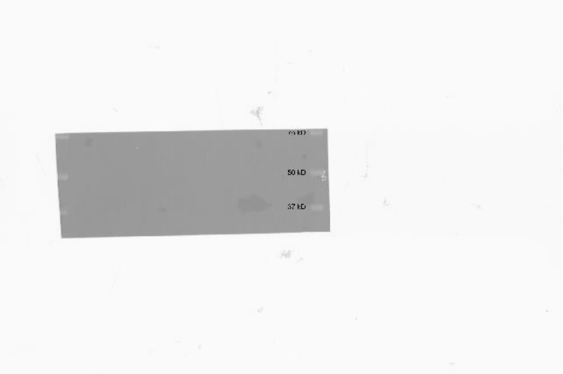

p-JNK Background

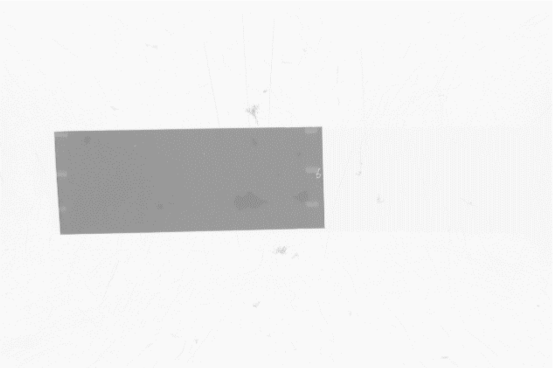

Supplement: Supplementary file 1 — Supplementary Figures. [file 41598_2022_18287_MOESM1_ESM.pdf]
